# A newborn with ambiguous genitalia and a complex X;Y rearrangement

**Published:** 2014-05

**Authors:** Mohammadreza Dehghani, Elena Rossi, Annalisa Vetro, Gianni Russo, Zahra Hashemian, Orsetta Zuffardi

**Affiliations:** 1 *Department of Molecular Medicine, University of Pavia, Pavia, Italy.*; 2 *Research and Clinical Center for Infertility, Shahid Sadoughi University of Medical Sciences, Yazd, Iran.*; 3 *Biotechnology Research Laboratories, Fondazione IRCCS Policlinico San Matteo, Pavia, Italy.*; 4 *Department of Pediatrics, Endocrine Unit, University Vita-Salute, San Raffaele Hospital, Italy.*; 5 *Shahid Sadoughi Hospital, Shahid Sadoughi University of Medical Sciences, Yazd, Iran.*

**Keywords:** *Ambiguous genitalia*, *46*, *XX testicular DSD*, *Inverted duplication and Xp terminal Deletion(Invdup del)*, *Rearrangement*, *Array Comparative Genomic Hybridization*, *FISH*

## Abstract

**Background:** In most mammals, sex is determined at the beginning of gestation by the constitution of the sex chromosomes, XY in males and XX in females.

**Case: **Here we report an interesting case characterized by ambiguous genitalia and ovotestis in a newborn carrying an apparently female karyotype (46 XX). Array Comparative Genomic Hybridization (Array-CGH) revealed an unbalanced rearrangement resulting in the deletion of the distal Xp and the duplication of the proximal Xp contiguous region with presence of the Y chromosome from Ypter to Yq11. Fluorescent in situ hybridization (FISH) showed that this portion of the Y was translocated to the tip of the abnormal X and that the duplicated portion of chromosome X was inverted. Altogether, the abnormal chromosome was a dicentric one with the centromere of the Y chromosome apparently inactivated.

**Conclusion:** The presence within the translocated Y chromosome of the SRY gene explains the devolopment of testes although it is not clear the reason for the genitalia ambiguity.

## Introduction

In mammals, sex is determined at the early gestation according to the constitution of the sex chromosomes, XY in males and XX in females (1). The Y chromosome is responsible for the development of testis thanks to the SRY gene at distal Yp whose role in sex determination is to initiate testis rather than ovary development from early bipotential gonads ( 1-3 ).

Sex development can be divided into two sequential processes: sex determination, in which the undifferentiated gonadal primordium commits to developing into either testes or ovaries; and sex differentiation, in which gonadal hormones act on the internal and external genitalia, resulting in differentiation into sexually dimorphic reproductive structures (1, 4, 5). Disorders of Sexual Development (DSDs) in humans are characterized by a complete or partial mismatch between the genetic sex and the phenotypic sex. Collectively, DSDs occur in at least 1 in 100 live births and include relatively mild forms such as hypospadias (1/500 births) as well as more severe conditions such as ambiguous genitalia (1/4,500 births) and complete sex reversal (46, XY females and 46, XX males; 1 in 20,000 births). Interestingly, DSDs account for 7.5% of all birth defects (2, 6-9).

46, XX testicular DSDs is the new term and classification of XX males or XX sex reversal (10). Based on the person’s phenotype or the genetic cause of 46, XX testicular DSDs, two classification are recognized, the first includes three clinical categories: a) XX males with normal genitalia, b) XX males with ambiguous genitalia, and c) XX true hermaphrodites with ovarian and testicular tissues. XX males can be divided into two molecular categories, according to the presence or absence of the Y-chromosome sequences (12-14). In 46, XX testicular DSD, individuals with two X chromosomes in each cell, which is the pattern that is normally found in females, have a male appearance. 

In most cases (80%), individual with this disorder have male external genitalia and, after puberty, develop normal pubic hair and normal penile size, but small testes, gynecomastia, and sterility resulting from azoospermia. Approximately 20% of the 46,XX testicular DSD individuals have ambiguous genitalia at birth ranging from cryptorchidism or hypospadias to a frank ambiguity making difficult gender assignment. In these cases gonads are constituted by ovotestis or mixed gonadal dysgenesis with ovarian-like structures at one side and testis differentiation at the other one. 

Although most of the XX DSD subjects suffer from a condition only related to abnormal gonads/genitalia, some of them present with a complex syndrome as it is the case of the palmoplantar hyperkeratosis with squamous cell carcinoma of skin and 46,XX sex reversal (OMIM #610644) due to autosomal recessive mutations in the RSPO1 gene (15). To date, many cases of XX males with or without SRY and apparently with no other Y-chromosome sequences have been reported ( 14). Approximately 85% of the XX testicular DSD cases are phenotypically male with unambiguous male genitalia at birth and are SRY positive. Most of these individuals and are not diagnosed until that puberty fails to proceed normally (16, 17). Usually they have a shorter-than-average stature (mean height: 168.2 cm, compared to normal mean height: 173.5 cm), gynecomastia, small testes, and azoospermia (        18 ). They rarely present with atypical genitalia (19-22). 

The remaining 15% of individuals with XX testicular DSD have ambiguous genitalia and SRY is positive in only a minority of the individuals (16,         23 ). Here we presented the case of a newborn ascertained because of ambiguous gentalia and some dysmorphic features.

## Case report

New born is only child of non-consanguineous couple. The first pregnancy ended in a spontaneous abortion. At birth the proband had normal stature and normal weight with 9/10 Apgar. At clinical examination we found forehead prominence, hypertelorism, saddle nose, thin lips, low set and small ear and bilateral clinodactyly of fifth fingers. The clitoris was hypertrophic and in the inguinal region two small masses were detected. A unic urethral meatus was present below two hypotrophic labia minora. Scrotum-like labia majora and hypospadias were also present. In echosonography gonads were not present in pelvic. The inguinal masses were removed and their histology revealed that they were ovotestes. 

**Figure 1:**
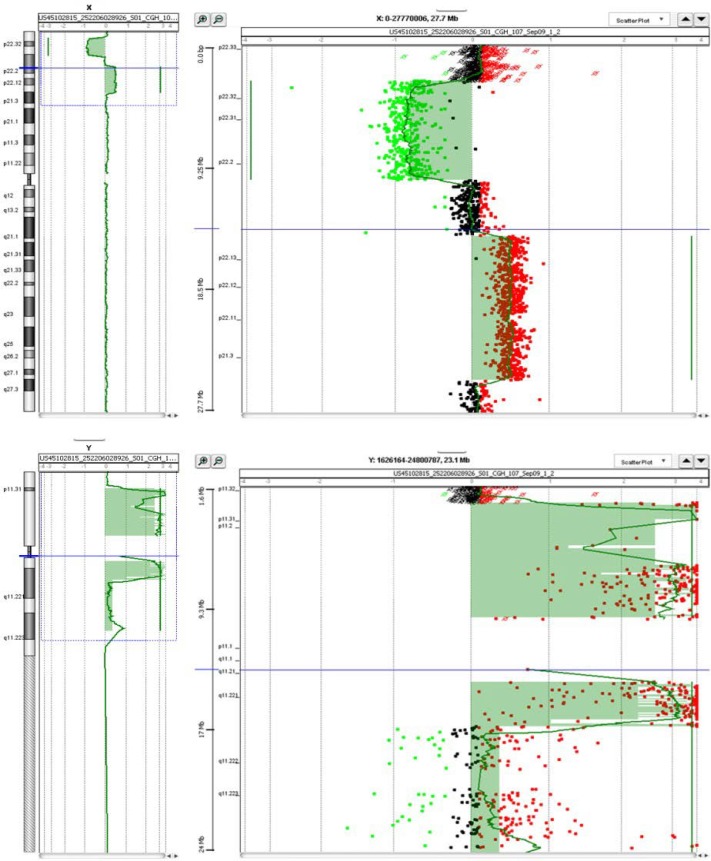
oligonucleotide aCGH platforms (180K SurePrint G3 Human Kit, Agilent Technologies, Santa Clara, CA) showed a complex rearrangement with the deletion of the distal Xp of about 10 Mb between Xpter and Xp22.2, and a 19.9Mb duplication of a proximal Xp contiguous region; the Xp region between 10227817-15816305 was normal; a 16.8 Mb portion of the Y chromosome was also present consisting in the entire short arm and a part of long arm with breakpoint in Yq11.22

PHA-stimulated lymphocytes cultures were set up from peripheral blood from the patient and the parents and the analysis was done on GTG banded metaphases, according to established guidelines. The karyotype was 46, XX with one short arm of the X chromosomes slightly longer. The karyotype of the parents was normal. Array-CGH analysis was performed by using oligonucleotide aCGH platforms (180K SurePrint G3 Human Kit, Agilent Technologies, Santa Clara, CA), as reported elsewhere (23). A 46, XX reference DNA (NA15510, Coriell Cell repositories) was used for all experiments. Changes in DNA copy number at a specific locus were observed as the deviation of the log2ratio value from 0 of at least three consecutive probes, by using Genomic Workbench v. 5.0.14 software (Agilent, ADM-2 algorithm with a threshold of 5). Oligomer positions refer to the Human Genome GRCh37 (hg19) assembly. Array-CGH analysis showed a complex rearrangement with the deletion of the distal Xp of about 10 Mb between Xpter and Xp22.2, and a 19.9Mb duplication of a proximal Xp contiguous region; the Xp region between 10227817-15816305 was normal; a 16.8 Mb portion of the Y chromosome was also present consisting in the entire short arm and a part of long arm with breakpoint in Yq11.22 (Figure 1). The final definition was: 

Arr Yp11.32q11.22 (11091-16824972) x1, Xp22.33p22.2 (61091-10207876) x1, Xp22.2p21.3 (15,834,910-25,337,964) x3

**Figure 2:**
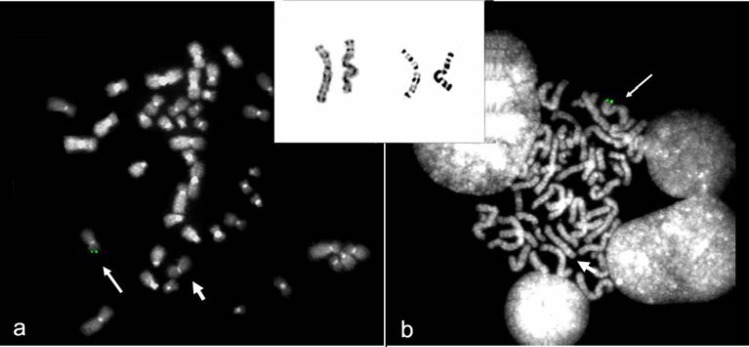
**a**: FISH analysis with BAC RP11-294K6 (6,503,905-6,527,019 Mb) confirmed the Xp22.33p22.3 deletion identified by array-CGH. **b**: FISH analysis with BAC RP11-639O7, covering the SRY gene, demonstrated that the portion of the Y chromosome translocated to the tip of the abnormal chromosome X contains the SRY gene. The short arrows indicate the derivative X. In the box above a cut out of chromosomes X is shown; on the right the derivative X

**Figure 3:**
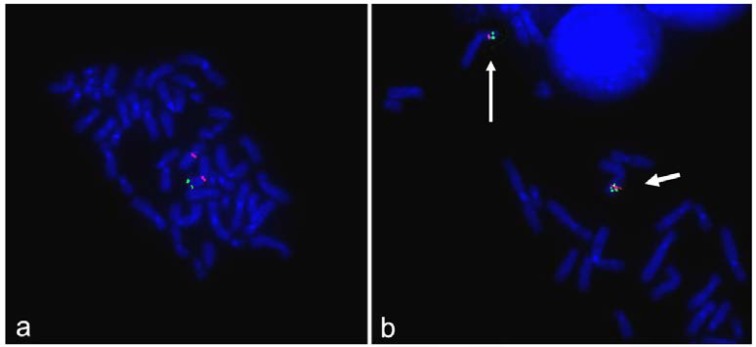
**a**: FISH analysis with alphoid probes specific for chromosome X (red) and for chromosome Y (green) showed that the Y centromere has clearly detached signals suggesting its inactivation. **b**: FISH analysis with BACs within the Xp duplicated region demonstrated that the duplication is inverted : RP11-124B4 (green) in Xp22 at 14,955,137-15,105,389 Mb and RP11-171N17 (red) in Xp22 at 21,969,414-22,248,218 Mb. The short arrow indicates the derivative X

To confirm array-CGH results and demonstrate the inversion of Xp duplicated region, FISH experiments were performed. All BAC probes used were selected according to the UCSC Human Genome Browser and were obtained from the human library RPC1-11. Alphoid probes were kindly provided by Mariano Rocchi (http://www.biologia.uniba. it/rmc/). X-inactivation analysis was performed according to Allen *et al* (24). Fluorescent in situ hybridization (FISH) confirmed the Xp deletion, demonstrated that the portion of the Y was translocated to the tip of the abnormal chromosome X and does not contain SRY and that the duplicated portion of chromosome X was inverted (Figure 2 and 3). Altogether, the abnormal chromosome was a dicentric one with the centromere of the Y chromosome apparently inactivated. 

X chromosome inactivation studies revealed that the derivative X was inactivated in all the 96 metaphases analyzed (data not shown).

## Discussion

Approximately 20% of the 46, XX testicular DSD individuals have ambiguous genitalia at birth ranging from cryptorchidism or hypospadias to a frank ambiguity making difficult gender assignment. In these cases gonads are constituted by ovotestis or mixed gonadal digenesis with ovarian-like structures at one side and testis differentiation at the other one. Here we presented a case with ambiguous genitalia at birth and inguinal mass with histology of ovotestis after surgery.

In most of the XX males *SRY *is transposed to the tip of Xp as a consequence of a recurrent Xp;Yp translocation arising predominantly by non-allelic homologous recombination between of *PRKX* and *PRKY* in a particular Y chromosome background consisting in a polymorphic cryptic inversion (25, 26). In our case, the *SRY* gene is indeed located at the end of one X chromosome but the rearrangement is completely different and much more complex leading to a classical inv dup del (Xp) in which the absence of the short arm telomere has been repaired the acquirement of a portion of the Y chromosome containing its centromere (27, 28). As a result, the rearranged chromosome was dicentric although the Y centromere appears inactivated according to the finding of two alphoid spots always detached (Figure 2a). We may speculate that originally the dicentric was unstable with loss of the Y chromosome portion in some cells. This hypothesis might explain the presence in the proband of ambigous genitalia otherwise incomprehensible. This rearrangement is sporadic and therefore this condition doesn’t have recurrent risk.

Our proband is a newborn and so far presents minor dysmorphic signs as forehead prominence, hypertelorism, saddle nose, thin lips, low set and small ear and bilateral clinodactyly of 5^th^ fingers. It seems likely that the deleted and duplicated portions of the X chromosome are responsible for them. The finding of the preferential inactivation of the abnormal X as stated in blood cells should avoid severe pathogenic consequences. This bodes well that the proband won’t develop a real medical condition. Certainly, although part of distal Xp is deleted, the proband should not have short stature being the two PAR regions containing the SHOX1 fully preserved. 
